# The activation thresholds and inactivation kinetics of poking-evoked PIEZO1 and PIEZO2 currents are sensitive to subtle variations in mechanical stimulation parameters

**DOI:** 10.1080/19336950.2024.2355123

**Published:** 2024-05-16

**Authors:** Nadja Zeitzschel, Stefan G. Lechner

**Affiliations:** Department of Anesthesiology, University Medical Center Hamburg-Eppendorf, Hamburg, Germany

**Keywords:** PIEZO1, PIEZO2, mechanotransduction, poking technique, patch-clamp, mechanobiology

## Abstract

PIEZO1 and PIEZO2 are mechanically activated ion channels that confer mechanosensitivity to various cell types. PIEZO channels are commonly examined using the so-called poking technique, where currents are recorded in the whole-cell configuration of the patch-clamp technique, while the cell surface is mechanically stimulated with a small fire-polished patch pipette. Currently, there is no gold standard for mechanical stimulation, and therefore, stimulation protocols differ significantly between laboratories with regard to stimulation velocity, angle, and size of the stimulation probe. Here, we systematically examined the impact of variations in these three stimulation parameters on the outcomes of patch-clamp recordings of PIEZO1 and PIEZO2. We show that the inactivation kinetics of PIEZO1 and, to a lesser extent, of PIEZO2 change with the angle at which the probe that is used for mechanical stimulation is positioned and, even more prominently, with the size of its tip. Moreover, we found that the mechanical activation threshold of PIEZO2, but not PIEZO1, decreased with increasing stimulation speeds. Thus, our data show that two key outcome parameters of PIEZO-related patch-clamp studies are significantly affected by common variations in the mechanical stimulation protocols, which calls for caution when comparing data from different laboratories and highlights the need to establish a gold standard for mechanical stimulation to improve comparability and reproducibility of data obtained with the poking technique.

## Introduction

PIEZO1 and PIEZO2 are mechanically activated ion channels that confer mechanosensitivity to various cell types [1–4]. PIEZO1, for example, is required for mechanotransduction in erythrocytes, vascular endothelial cells, and chondrocytes [5–9], whereas PIEZO2 is important for mechanotransduction in primary sensory afferents because it is involved in the detection of light touch, mechanical pain, proprioception, airway stretch, and bladder distension [10–16]. Moreover, PIEZOs contribute to the regulation of neurite outgrowth, wound healing, and tumor cell dissemination, probably by detecting traction forces acting on the plasma membrane during neurite extension and cell migration [17–20].

The functional properties of PIEZO channels are usually examined in patch-clamp recordings using either the pressure-clamp or the poking technique. In the pressure-clamp technique, PIEZO-mediated currents are recorded in cell-attached mode, and the channels are activated by stretching the membrane patch inside the patch pipette by applying negative pressure. In the poking technique, PIEZO currents are recorded in the whole-cell mode and activated by focal mechanical indentation of the plasma membrane with a small fire-polished patch pipette. The poking technique was first established by McCarter et al. and was later refined by Wood and Lewin labs [21–23], who used the technique to examine the back then unknown ion channel that mediates native mechanotransduction currents in cultured primary somatosensory neurons. Since the discovery of PIEZO channels in 2010 [1], the poking technique has become one of the most frequently used techniques, alongside the pressure clamp technique, for studying mechanosensitive ion channels, including PIEZO channels. The advantages and disadvantages, as well as the limitations and pitfalls of the two techniques, have been summarized and discussed in numerous review articles, including a recent example by Lewis and Grandl, which also provides important information about equipment calibration and data analysis [24–26]. However, an important point that has received little attention in previous studies is that subtle variations in the stimulation protocol may have profound effects on the outcome of patch-clamp recordings of PIEZO channel-mediated currents. This is particularly noteworthy because there is no common standard for mechanically stimulating the cell surface in the poking assay. A comparison of the mechanical stimulation protocols used by the leading laboratories in the field revealed that the mechanical stimuli that were used significantly differed with regard to the stimulation angle, size of the stimulation probe, and stimulation velocity. Thus, angles between 30° and 80° have been reported, and stimulation probes with tip diameters from 2 to 5 µm have been used in previous studies [1,20,25,27–34]. Moreover, the reported stimulation velocities range from 0.2 µm/ms to 5 µm/ms, and some researchers even increased the stimulation velocity as they increased the stimulus magnitude [27,33]. The latter, a relatively large range of stimulation velocities, is particularly concerning, considering that we have previously shown that stimulation velocity has a strong effect on PIEZO current amplitudes [20,35]. Moreover, during many years in which the poking technique has been used in our lab [13,14,20,35–40], anecdotal evidence has accumulated suggesting that the stimulation angle and tip diameter of the stimulation probe may also strongly affect the outcome of the experiments. Thus, we systematically investigated the impact of variations in stimulation velocity, stimulation angle, and stimulation probe size on the properties of the poking-evoked PIEZO1 and PIEZO2 currents.

## Materials and methods

### Cell culture

Mouse neuroblastoma Neuro2A PIEZO1-Knockout cells were a gift from Gary R. Lewin [41] and grown at 37°C and 5% CO2. Dulbecco’s modified Eagle’s medium and optimal minimal essential medium (Opti-MEM) (1:1 mixture) were supplemented with 2 mM L-glutamine, 10% fetal bovine serum, and 1% penicillin/streptomycin (all from Thermo Fisher) and used to grow the cells. For electrophysiological experiments, cells were seeded on acid-washed and poly L-lysine (PLL; Sigma)-coated glass coverslips one day prior to transfection.

### Transfection

One day after seeding on coverslips, N2A-P1KO cells were transfected with either PIEZO2-HA-IRES-GFP plasmid [35] or PIEZO1-mScarlet plasmid [20] with polyethylenimine (PEI, Linear PEI 25 K; Polysciences). For transfection of one coverslip in a well, 0.6 µg plasmid DNA was diluted in 20 µL PBS and then incubated with 16 µL of PEI mix (7 µL of 360 µg/ml PEI with 9 µL PBS) for at least 5 min. The DNA-PEI mix (35 µL) was then added slowly to one well while swirling gently to mix. After 24 h, the medium was replaced with a fresh medium. After 48 h, cells were used for patch-clamp recording.

### Electrophysiology

The mechanically activated currents were recorded at room temperature using an EPC10 amplifier with PatchMaster software (HEKA Elektronik). Patch and poking pipettes were produced from borosilicate capillaries that were pulled with a *p*-97 Flaming-Brown puller (Sutter Instrument, CA). The patch pipettes had resistances between 2 and 5 MΩ. Patch pipettes used for mechanical stimulation were fire-polished to completely close the tip and obtain tip diameters between 2 and 5.5 µm. The intracellular buffer with which the patch pipettes were filled contained the following (in millimolar): 125 potassium gluconate, 7 KCl, 1 CaCl2, 1 MgCl2, 10 HEPES, and 4 EGTA (pH 7.3 with KOH). The bath solution contained the following (in millimolar): 4 KCl, 140 NaCl 1 MgCl2, 2 CaCl2, 4 glucose and 10 HEPES (pH 7.4 with NaOH). Cells were clamped at a holding potential of −60 mV and mechanically stimulated with a series of 25 mechanical ramp-and-hold stimuli in 0.2 µm increments with a poking pipette driven by a *p*-840.20 preloaded piezo actuator (Physik Instrumente GmbH, Germany). The positioning of the poking probe was changed to three different angles (35°, 45°, and 70°) relative to the dish surface. To examine the velocity dependence, the poking probe was displaced at the same distance (8 µm), and the velocity of protrusion was halved after each step, starting at 2 µm/ms and reaching 0.062 µm/ms in the last stimulation.

The auto function of Patchmaster was used to compensate for pipette and membrane capacitance. The evoked whole-cell currents were recorded at a sampling frequency of 200 kHz and filtered with 2.9 kHz low-pass filter. Recordings with excessive leak currents or unstable access resistance were excluded from analysis. The mechanical thresholds of the PIEZO-mediated currents were determined by measuring the time at which the current passed the detection threshold (−10 pA) and determining the displacement of the poking probe at this time point. Time-to-peak analysis was used to determine the timespan between the current onset (the same as previously determined) and the time of the peak current amplitude. The inactivation time constants (τinact) were determined by fitting the mechanically activated currents to a single exponential function (C1 + C2*exp(−(t − t0)/τinact)), where C1 and C2 are constants, t is time, and τinact is the inactivation time constant.

### Statistics

All electrophysiological data were analyzed using custom written scripts in IgorPro 6.37 and IgorPro 8 (Wavemetrics). Results are expressed as mean ± SEM (unless otherwise noted). Statistical analyses were performed using the Prism 10 (GraphPad Software, Inc.). The data distribution was systematically evaluated using D’Agostino-Pearson, and the following statistical tests were chosen accordingly. The statistical tests that were used, the exact *p* values and information about the number of independent biological replicates are provided in each of the figures or in the corresponding legends.

## Results

**Figure 1.**
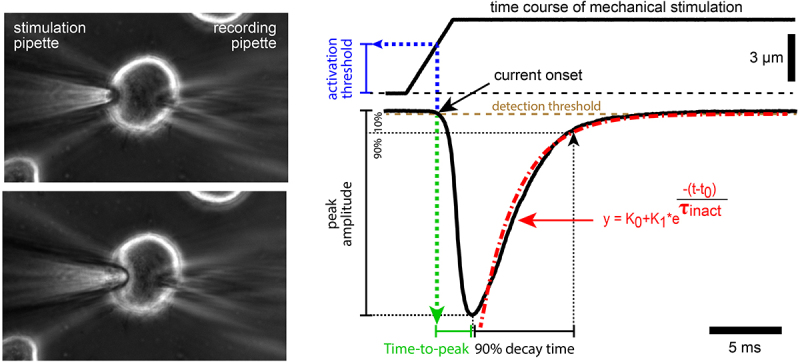
Experimental setup.

In whole-cell patch-clamp recordings, PIEZO1- and PIEZO2-mediated mechanotransduction currents are usually evoked by a series of mechanical ramp-and-hold stimuli of increasing magnitude that are applied to the cell surface using a fire-polished patch pipette (Figure 1). In response to such stimuli, both PIEZO1 and PIEZO2 generate inward currents with increasing peak amplitudes that inactivate quickly (within ~2–30 ms) before the hold phase of the stimulus ends (Figure 1). To characterize PIEZO-mediated currents, most researchers routinely analyze the stimulus–response curves (peak amplitude vs. stimulation magnitude), inactivation kinetics, which is usually fitted with a single exponential decay function, and mechanical activation threshold (i.e. the stimulus magnitude at which the currents are elicited; see annotations in Figure 1 for details). In addition, some researchers have analyzed the activation kinetics (e.g. time-to-peak, i.e. time from current onset to peak) and if the inactivation phase cannot be properly fitted with an exponential equation, the decay time (e.g. time from peak to 10% of peak amplitude, hereafter referred to as 90% decay time; Figure 1). Here, we investigated the extent to which these outcome parameters are altered by subtle variations in the mechanical stimulation protocol.

(a) Image of the whole-cell patch clamp setup on the target cell. (b) Schematic depiction of experimentally determined mechanically evoked current parameters.

### PIEZO1 but not PIEZO2 activates and inactivates faster at higher stimulation angles

**Figure 2.**
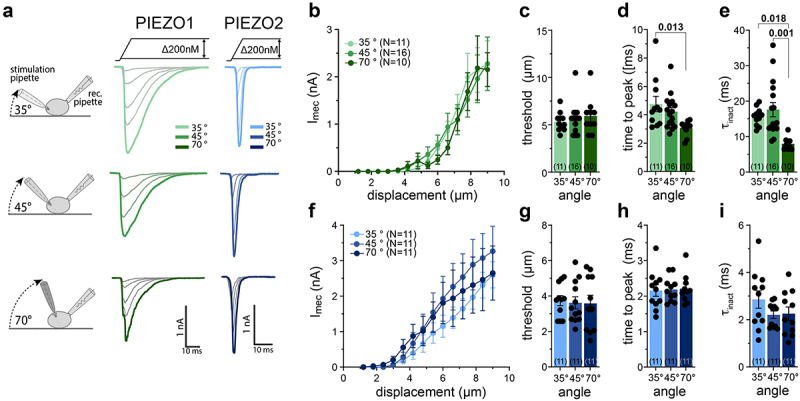
Poking-evoked PIEZO-mediated currents are regulated by the angle of the poking probe.

We first considered the impact of the angle at which the probe used for the mechanical stimulation was positioned relative to the bottom of the cell culture dish. To this end, we compared PIEZO1 and PIEZO2-mediated currents that were evoked at stimulation angles of 35°, 45°, and 70°, while the stimulation velocity (1.5 µm/ms) and the size of the stimulation probe (3 µm) were kept constant (Figure 2a). We did not test stimulation angles greater than 70°, even though 80° was used in some previous studies, because the spatial arrangement of our microscope did not allow the positioning of the glass rod at such high angles. We did not observe any differences in the stimulus response curves or mechanical activation thresholds of PIEZO1 and PIEZO2 at different stimulation angles (Figure 2b–g). However, significant differences were observed in the activation and inactivation kinetics of PIEZO1. Thus, the time-to-peak of PIEZO1 currents was reduced from 4.76 ± 0.54 ms at 35° and 4.21 ± 0.30 ms at 45° to 3.07 ± 0.17 ms at 70° (Figure 2d). The effect on the inactivation kinetics was even more pronounced, such that the inactivation time constant (τinact) was reduced by more than 50% when the glass rod was positioned at an angle of 70° (τinact = 7.98 ± 0.67 ms) as compared to 35° (τinact = 15.63 ± 0.76 ms) or 45° (τinact = 17.63 ± 1.98 ms, Figure 2e). For PIEZO2-mediated currents, we also observed a trend toward faster inactivation at higher stimulation angles (35°: τinact = 2.86 ± 0.37 ms, 45°: τinact = 2.21 ± 0.16 ms, 70°: τinact = 2.26 ± 0.28 ms), but no difference in the time-to-peak (Figure 2h,i).

(a) Cartoons illustrating the different angles used during the poking stimulation with example traces of mechanically evoked PIEZO1 and PIEZO2 currents with the depicted probing angles. (b) Displacement–response curves of poking-evoked currents recorded from N2A cells expressing PIEZO1. (c-e) Mechanical threshold, time to peak and inactivation time constant for PIEZO1 at different poking angles. (f) Displacement–response curve of PIEZO2 at different poking angles. (g-i) Mechanical thresholds, time to peak and inactivation time constants for mechanically evoked PIEZO2 currents. All data are represented as means ± SEM and the black circles in (c-e) and (g-i) represent the individual values from each recorded cell. The numbers of cells per group are indicated in the graph. Data shown in (c-e) and (g-i) were compared using one-way ANOVA followed by Tukey’s multiple comparison test. For P-values smaller than 0.05, the exact values are provided above the bars.

### PIEZOs inactivate faster when cells are poked with larger glass rods

**Figure 3.**
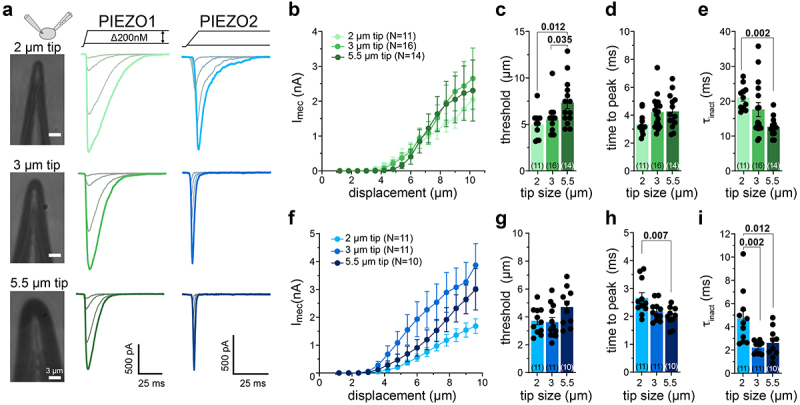
Poking-evoked PIEZO-mediated currents are regulated by the size of the poking probe.

Next, we asked whether variations in the size of the stimulation probe alter the outcome of the PIEZO current recordings. The exact size of the stimulation probe is commonly not provided in the methods section of research papers describing PIEZO channel recordings. Instead, researchers provide a range that usually lies between 2 and 5 µm probe tip diameter. Thus, we compared PIEZO1 and PIEZO2 currents evoked by three different stimulation probes with tip diameters of 2, 3, and 5.5 µm (Figure 3a). We did not observe significant differences in the current amplitudes at supra-threshold stimulation (Figure 3a,b,f), but observed a significant increase in the mechanical activation threshold of PIEZO1 with increasing probe diameter (Figure 3c). We observed a similar yet non-significant trend in the activation thresholds of PIEZO2 (Figure 3g). Most importantly, we observed a strong correlation between the tip diameter and inactivation kinetics for both PIEZO1 and PIEZO2. Thus, PIEZO1 currents evoked with a small probe (2 µm diameter) inactivated significantly slower than currents evoked with larger tips (2 µm tip, τinact = 21.07 ± 1.04 ms; 3 µm tip, τinact = 17.63 ± 1.98 ms; 5.5 µm tip, τinact = 12.69 ± 0.77 ms; Figure 3e). Likewise, PIEZO2 currents evoked with a 2 µm probe exhibited inactivation time constants of 4.71 ± 0.70 ms, whereas PIEZO2 currents evoked with 3 µm and 5.5 µm probes had inactivation time constants of 2.21 ± 0.16 ms and 2.61 ± 0.40 ms, respectively (Figure 3i). The activation kinetics of PIEZO2, but not of PIEZO1, was also dependent on the stimulation tip size, such that the time-to-peak decreased from 2.66 ± 0.19 ms for currents evoked with a 2 µm tip to 2.19 ± 0.10 ms and 1.99 ± 0.11 ms for currents evoked with 3 µm and 5.5 µm tips, respectively.

(a) Images of poking probes with example traces of currents evoked by mechanical ramp-and-hold stimuli in cells expressing PIEZO1 or PIEZO2. (b) Displacement–response curves of PIEZO1-mediated currents evoked by poking probes with the indicated tip diameters. (c–e) Mechanical activation thresholds, inactivation time constants, and time-to-peak of PIEZO1 currents evoked by the indicated poking probes. (f) Displacement–response curves of PIEZO2-mediated currents evoked by poking probes with the indicated tip diameters. (g-i) Mechanical activation thresholds, inactivation time constant, and time to peak of PIEZO2 currents evoked by differently sized poking probes. All data are represented as mean ± SEM, and the black circles in (c-e) and (g-i) represent the individual values from each recorded cell. The number of cells per group is shown on the graph. Data shown in (c-e) and (g-i) were compared using one-way analysis of variance (ANOVA) followed by Tukey’s multiple comparison test. For P-values smaller than 0.05, exact values are provided above the bars.

### The mechanical activation threshold of PIEZO2 decreases with increasing stimulation speeds

**Figure 4.**
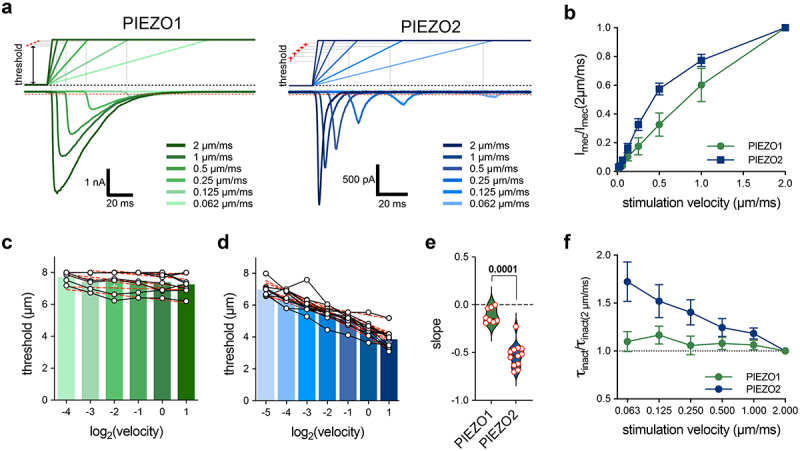
PIEZO channels are sensitive to stimulation velocity.

Finally, we investigated the stimulation velocity dependence of PIEZO1 and PIEZO2. To this end, we kept the stimulus magnitude, probe size (3 µm), and positioning angle (45°) of the stimulation probe constant and stepwise reduced the stimulation velocity by a factor of 2 starting from 2 µm/ms. Consistent with observations from our previous work [20], we found that the peak amplitudes of both PIEZO1 and PIEZO2 currents decreased as the stimulation velocities decreased (Figure 4a,b). Interestingly, however, the mechanical activation thresholds of PIEZO1 did not differ at different stimulation velocities (Figure 4c), while the thresholds of PIEZO2 showed a clear correlation with stimulation velocity, in that the thresholds were reduced with increasing stimulation velocity (Figure 4d). To directly compare the velocity dependences of the mechanical thresholds of PIEZO1 and PIEZO2, we fitted the velocity vs. threshold data of each cell with a simple linear regression model (red dashed lines in Figure 4c,d), which revealed that the activation threshold of PIEZO2 was reduced by 0.53 ± 0.04 µm when the stimulation velocity was increased by a factor of 2 (Figure 4e). The thresholds of PIEZO1 also exhibited some degree of velocity dependence, which was, however, less pronounced (~0.11 ± 0.03 µm per doubling of velocity, Figure 4e). Notably, we also observed a decrease in the inactivation kinetics of PIEZO2, but not PIEZO1, at low stimulation velocities (Figure 4f). In summary, our data show that the stimulation velocity is a major determinant of the peak current amplitude and, in the case of PIEZO2, of the mechanical activation threshold and inactivation time constant.

(a) Example traces of currents evoked by mechanical ramp-and-hold stimuli with the indicated ramp speeds in cells expressing PIEZO1 or PIEZO2. (b) Comparison of the velocity sensitivities of PIEZO1 and PIEZO2. Graph shows the mean normalized current amplitudes ± SEM as a function of stimulation velocity. (c) PIEZO1 thresholds at different stimulation velocities with linear regression in dotted red line. (d) Mechanical threshold of PIEZO2 at different stimulation velocities with linear regression (dotted red line). White circles in (c and d) represent individual values from each recorded cell and data points from the same cell are connected with a black line (e) Violin plot with the slopes from the regression curves shown in graphs (c) and (d) of PIEZO1 and PIEZO2 with individual values. (f) Normalized inactivation time constants of PIEZO1 and PIEZO2 are dependent on the stimulation velocity. Data are presented as mean ± SEM. Comparison with one-way ANOVA with Tukey’s post test. *p* values are indicated in the graphs. The number of measured cells for all graphs was *N*=8 for PIEZO1 and *N*=13 for PIEZO2.

## Discussion

Mechanical ramp-and-hold stimuli used to elicit PIEZO-mediated currents in patch-clamp recordings are not only defined by their amplitude but also by (i) the speed at which the probe moves (ramp speed), (ii) the contact area between the stimulation probe and the cell surface (probe tip size), and (iii) the angle at which the probe is positioned. Here, we systematically examined the impact of variations in these three parameters on the sensitivity, amplitude, and kinetics of poking-evoked PIEZO1 and PIEZO2 whole-cell currents.

The most prominent observation was that the mechanical activation threshold of PIEZO2 decreased with increasing stimulation velocities, whereas the threshold of PIEZO1 did not change within the examined range of stimulation velocities. Before PIEZO channels were discovered, the amplitudes of the native rapidly adapting mechanotransduction current in dorsal root ganglion neurons, which later turned out to be mediated by PIEZO2 [10,13], were shown to be dependent on mechanical stimulation velocity [42]. Back then, this velocity dependence was proposed to result from the fast inactivation of the channel, which supposedly causes a considerable fraction of the channels to close before the mechanical stimulation reaches its maximum, resulting in smaller peak current amplitudes. Considering that the peak current amplitudes of PIEZO1 decline even more with decreasing stimulation velocities (Figure 4b), although PIEZO1 inactivates much slower than PIEZO2 (Figures 2 and 3;[1]), this hypothesis seems unlikely. An alternative explanation for reduced sensitivity at slow velocities is that cellular remodeling or adaptation may more efficiently compensate for the mechanical strain induced by slow mechanical stimuli. Such adaptive processes would, however, equally affect both PIEZO1 and PIEZO2 and can thus only explain the reduction in peak current amplitudes, which was observed for both channels (Figure 4b), but not the velocity dependence of the activation threshold, which is specific for PIEZO2 (Figure 4c–e). The most likely explanation for the differential velocity dependence of the activation thresholds of PIEZO1 and PIEZO2 is that the two channels are activated by different intracellular force-coupling mechanisms, and that the mechanism by which forces exerted on the cell surface are transmitted to PIEZO2 is less efficient at slow stimulation velocities. Indeed, there is evidence suggesting that PIEZO1 is predominantly activated by force-from-lipid force transmission (i.e. direct gating by changes in membrane tension), whereas PIEZO2, at least in the poking assay, is mainly activated by force-from-filaments (i.e. mechanical stimuli applied to the cell surface are transmitted to the channel via the cytoskeleton) [43]. Hence, slow mechanical stimuli are more efficiently dampened by dynamic cytoskeletal reorganization, which could explain why larger stimuli are required to activate PIEZO2 but not PIEZO1 at slow stimulation velocities. This is, however, pure speculation and additional experiments, which are beyond the scope of this study, will be required to address this interesting question. Irrespective of the underlying mechanism, the observation that the activation threshold of PIEZO2 is velocity-dependent is very interesting because it provides an explanation for the velocity sensitivity of sensory afferents that detect vibrotactile stimuli (rapidly-adapting Aβ-fiber low-threshold mechanoreceptors), which require PIEZO2 for mechanosensitivity [10,13] and are also most sensitive to tactile stimuli impinging on the skin with velocities of approximately 1 µm/ms [44,45].

We also observed a correlation between the stimulation probe size and inactivation time constant, as well as a correlation between the stimulation angle and inactivation time constant for PIEZO1 and PIEZO2. In this context, it is important to note that although we and others commonly use the term “inactivation” to describe the current decay kinetics, it is actually misleading. Inactivation is an inherent property of PIEZO1 and PIEZO2, which is determined by intramolecular interdomain interactions [28,46,47] and can be further modulated by interacting proteins, such as the MyoD family inhibitor proteins (MDFIC, MDFI) [48] and TMEM150c [49], as well as by changes in the pH, temperature, and lipid composition of the plasma membrane [50]. The current decay during sustained mechanical stimulation, the time course of which is fitted to obtain the inactivation time constant, results not only from channel inactivation but also from stimulus adaptation. Thus, due to relaxation of the stimulated cell during sustained mechanical stimulation, membrane tension, and cytoskeletal strain, and consequently the local force acting on the channel, declines in a time-dependent manner, which inevitably contributes to the current decay kinetics and thus to what we and others commonly refer to as the inactivation time constant. Since inactivation is an inherent property of PIEZO channels, it cannot be altered by changes in the stimulation angle or the size of the stimulation probe. Thus, the most likely explanation for the observed dependency of the current decay kinetics is that the cell relaxation and/or the force dispersion time courses change with angle and probe size.

To test this hypothesis, force-dispersion within the cell in response to focal mechanical stimulation of the plasma membrane would have to be examined experimentally, but this was far beyond the scope of this study, the main goal of which was to create an awareness for potentially confounding factors in the poking assay. Hence, we can only speculate about intracellular force-transmission pathways and about how they may differ in different stimulation scenarios. As mentioned earlier, PIEZOs can be activated by force-from-lipids (FFL; i.e. conformational changes that result in channel activation are triggered by membrane stretch-induced changes in lipid bilayer asymmetry) as well as by force-from-filament (FFF; i.e. forces that are transmitted to the channel via tethers that physically link the channel to the cytoskeleton or the extracellular matrix) (see [19,24,43,51,52] for excellent reviews on this topic). Whether these two force-coupling mechanisms act synergistically or independently of each other to activate PIEZOs is, however, still unclear. Considering the existence of two different force-coupling mechanisms and considering further that poking of the cell surface inevitably induces both membrane stretch as well as cytoskeletal strain, it is tempting to speculate that the whole-cell currents that are evoked by such stimuli might be mediated by different pools of channels that are either activated by different mechanisms or experience different ratios of FFF and FFL. Thus, since membrane tension is only propagated a short distance across the cell surface [53], sufficient membrane stretch and thus FFL might only be exerted on channels that are located in close proximity to the site of stimulation, whereas channels that are located further away could predominantly be exposed to forces transmitted by the cytoskeleton (FFF). Accordingly, an increase in the size of the stimulation probe would increase the cell surface area that experiences stretch, which could change the proportion of channels that are activated by FFL and thus the kinetics and threshold of poking evoked currents. In addition to the force that is exerted by the stimulation probe itself, the patch pipette and the surface of the culture dish, which are located on opposite sides, inevitable generate counteracting forces that result in local cell deformation and membrane stretch. Considering the spatial arrangement of these three elements (see e.g. cartoon in Figure 2a), it seems likely that changes in the stimulation angle would also alter the magnitude of the opposing forces that originate from the patch pipette and the dish surface, which may – analogous to changes in the probe diameter – change the ratio of channels that are activated by FFF and FFL and thus alter the kinetics and thresholds of the poking evoked currents.

Although our data do not allow definitive conclusions regarding the mechanistic basis of the velocity-sensitivity of PIEZO2 and the mechanisms underlying the probe size and angle dependence of inactivation kinetics, our study demonstrates that subtle changes in any of these three stimulation parameters can have profound effects on key characteristics of PIEZO-mediated currents. Thus, stimulation velocity, probe size, and stimulation angle should be carefully considered and controlled when designing patch-clamp experiments for studying PIEZO2 and should, most importantly, be accurately reported when publishing data to improve comparability and reproducibility of PIEZO-related data obtained with the poking technique.

## Data Availability

The data that support the findings of this study are available from the corresponding author upon reasonable request.
